# Micronutrient treatment for children with emotional and behavioral dysregulation: a case series

**DOI:** 10.1186/s13256-015-0735-0

**Published:** 2015-10-29

**Authors:** Bonnie J. Kaplan, Paula Hilbert, Ekaterina Tsatsko

**Affiliations:** 1 Faculty of Medicine, University of Calgary, The Child Development Centre, 3820 - 24 Avenue NW, Calgary, AB T3B 2X9 Canada; 2 59 Dogwood Trail, Ocala, FL 34472 USA; 3 1501, 1410 1 St SE, Calgary, AB T2G 5 T7 Canada

**Keywords:** Micronutrients, Treatment outcome, ADHD, Child behavior disorders

## Abstract

**Introduction:**

In clinical studies of adults and children, broad-spectrum micronutrients (minerals and vitamins) have proven beneficial for improving mood regulation and attention. We report here pilot work whose primary objective was to evaluate the feasibility of studying micronutrient treatment in school-aged children with emotional and behavioral problems. Issues examined included feasibility of participant recruitment from a culturally diverse population, probability of sample retention for a 12-week trial, acceptability of the outcome measures, supplement adherence, as well as trends in treatment benefit.

**Case presentation:**

The families of two boys (ages 5 and 6) and one girl (age 14) were invited to participate in a 12-week pilot trial of micronutrients carried out during the summer months. All children were enrolled in the private school at which future research was being considered. During the previous school year, all three had been extremely difficult to educate due to their inability to pay attention and learn, as well as their behavior problems. Although the two younger children had not been formally diagnosed, parents and teachers provided reports of hyperactivity and inability to focus on education in the classroom. The oldest child was often aggressive, and had been diagnosed with bipolar disorder, attention deficit hyperactivity disorder, and oppositional defiant disorder. All three children were Hispanic and spoke both Spanish and English. For 12 weeks, after signing consent forms, the children’s parents provided weekly ratings on the parent-report Child Mania Rating Scale; the children consumed the micronutrient formula daily and provided a daily rating of how they felt. The parent ratings revealed significantly improved behavior, *p* = .002. Children’s ratings approached the ideal level of 7, indicating “happy” self-reports. Parent interviews confirmed the weekly scores. Several feasibility questions were answered: all three children completed the 12-week trial, all scores were completed by parents and children, adherence to the protocol was excellent, and no adverse reactions emerged.

**Conclusions:**

Family physicians and pediatricians are often confronted with the challenge of improving the lives of families whose children experience school crises due to emotional and behavioral dysregulation. Three children, who participated in pilot work to determine the feasibility of further investigations, experienced impressive changes that clearly warrant both research and clinical exploration.

## Introduction

Success in the classroom is a function of many variables, not the least of which is children’s ability to regulate emotions, attention and behavior. Even very intelligent children who lack this self-regulation may struggle in school, and may be faced with multiple disciplinary incidents or even expulsion.

The Learning Curve Academy in north central Florida is an independent school established in 2007. It is an inclusion school and serves typically developing children as well as disabled children and children diagnosed with learning and behavioral problems. Approximately 50 children are enrolled in grades kindergarten to grade 12 in this private school, and many of them receive specialized services. Some of the children have been diagnosed with a behavioral/mental disorder, most frequently attention deficit hyperactivity disorder (ADHD) and bipolar disorder. Most had previously failed in the public school system, where assessments by social workers and psychologists resulted in Individual Educational Plans (IEPs). The tuition fees for all three children were heavily subsidized by the state government because of the severity of their learning and behavioral problems, or because their families live at or below poverty level. Although school staff members focus primarily on education and fulfillment of IEP goals, the significant emotional and behavioral problems in the classroom often prevent successful achievement.

In the last 15 years, several studies have demonstrated that nutrient supplementation for school-aged children can improve their attention and emotional self-regulation, as well as decrease behavioral problems and disciplinary events. Schoenthaler showed that giving 40 schoolchildren aged 6–12 years a broad spectrum of nutrients resulted in 47 % fewer antisocial acts requiring discipline than the 40 children who received a placebo [1]. Outside the classroom, research by Harding and colleagues showed improved self-regulation in children diagnosed with ADHD or bipolar disorder who were treated with minerals and vitamins [2]. In fact, the improvements in ADHD symptomatology in 20 children given a range of dietary nutrients over a 4-week period were similar in magnitude to the changes obtained from methylphenidate. A randomized, placebo-controlled trial (RCT) by Katz *et al.* evaluated children diagnosed with ADHD: half received an herbal compound with essential nutrients, including essential fatty acids, phospholipids, essential amino acids, B vitamins, vitamins E and C, and minerals, and half received a placebo [3]. Change over the 4-month trial period was assessed with the Test of Variables of Attention (TOVA) [3]. The children taking the active ingredients exhibited significant improvements on all TOVA subscales whereas those taking the placebo did not change.

These studies are intriguing, but it is difficult to generalize from any of them as the nutrient treatments have varied so much across investigations. However, there are now several reports using a single broad-spectrum formula of minerals and vitamins (EMPowerplus^1^) in children with emotional and behavioral dysregulation. Kaplan and colleagues conducted an open-label trial of this formula with children with a variety of psychiatric disorders, including ADHD, bipolar disorder, anxiety, and oppositional defiant disorder [4]. Six of the 11 children in the trial met criteria for ADHD, one of whom dropped out. After 16 weeks of taking the nutrient formula, and being monitored with the Child Behavior Checklist (CBCL), the ten remaining children were significantly improved on seven CBCL scales: withdrawn behavior, anxious/depressed mood, social problems, thought problems, attention problems, delinquent behavior, and aggressive behavior. Later, Rucklidge *et al.* published a database analysis of 41 children with ADHD taking this same formula, finding a significant improvement in ADHD symptoms [5]. The improvement in behavior ratings was 47 % and was sustained over a 6-month period. Frazier *et al.* used the same broad-spectrum formula in a small group of children who met criteria for pediatric bipolar disorder, most of whom also had ADHD [6]. This 8-week trial resulted in significant improvements in emotion regulation. Finally, in adults, Rucklidge and colleagues reported a positive RCT in those with ADHD treated for 8 weeks with the same micronutrient formula [7]. This study included 80 adults with ADHD, half randomized to micronutrients and half to placebo. Those who received the micronutrient treatment reported greater change in the key areas of ADHD symptoms (attention, hyperactivity and impulsivity) than those who received the placebo. Of the approximately 25 % of the participants who entered the trial depressed, greater benefit was found in those treated with the micronutrients compared to placebo.

As there is now an increasing body of literature on this one micronutrient formula, enabling comparisons and generalizations across studies, we decided to revisit the classroom environment studied by Schoenthaler over 15 years ago, and determine whether this formula should be studied systematically for its potential benefit for self-regulation in schoolchildren. Prior to doing so, it was important to evaluate several issues related to feasibility of pursuing this line of research, including feasibility of participant recruitment from a culturally diverse population, probability of sample retention for a 12-week trial, acceptability of the outcome measures, and supplement adherence. The results of this feasibility pilot study are reported here, including the trends in treatment response.

## Case presentation

### Participants

The three children who participated in this pilot study were ones who had had extremely challenging problems with classroom attention and behavior during the previous school year, happened to be available during the summer months, and were willing to participate. None was receiving any other treatments or interventions during their participation in this pilot study.

At the time of the trial, the three children were aged 5 (male), 6 (male), and 14 (female) years. All three were Hispanic (which is the majority of this school’s population) and spoke both Spanish and English. All materials were translated for their parents, who were not always fluent in English. None of the three children had any known physical health problems, and none were taking medications. The two younger children had not been formally diagnosed, in both cases their classroom difficulties with attention and behavior regulation had been significant challenges during the previous school year. The 14-year-old girl had been diagnosed with bipolar disorder, ADHD, oppositional defiant disorder, and aggressive behavior but was not taking psychiatric medication.

### Measures

Two measures were used to monitor symptoms over the 12 weeks. Parents were asked to complete a weekly checklist consisting of the 21 items from the parent-report Child Mania Rating Scale [8]. This form was labeled the “Parent Weekly Form” for current purposes, and covered topics such as irritability, sleep, energy levels, risky behavior, and rapid mood changes. Each item is scored from 0 (not at all) to 3 (very often), yielding a maximum weekly score of 63.

For the children, a short checklist was developed and was used daily. They were asked to rate the entire day on a nominal categorical scale: 1 (happy), 2 (sleepy), 3 (sad), or 4 (mad). A verbal version was used for the older child; a version that used happy and angry faces was employed for the two younger children. Since it was a daily score, the checklists were simply summed for a total weekly score.

In addition, the study coordinator met with each family every 2 weeks to ensure compliance with capsule consumption, as well as questionnaire completion. In that interview, she also questioned families about any adverse events.

### Procedure

Dose was titrated according to the following schedule: one capsule/day for the first week, two/day for the second week, three/day for the third day, and then four/day from week 4 until the end of the study (week 12). Beginning in the second week, the doses were divided into morning and afternoon, so for most of the 12-week trial each child consumed two capsules twice a day.

### Results

**Fig. 1:**
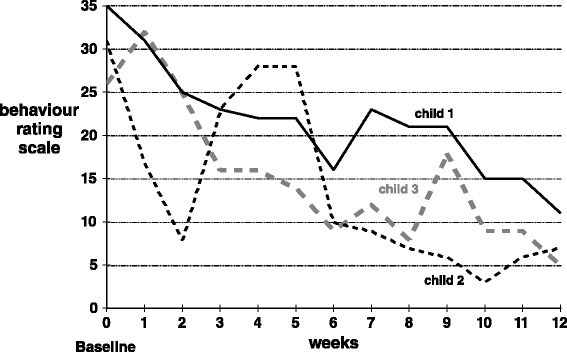
Parent-reported scores. Each weekly score could range from 0 to 63, with 0 representing virtually no behavioral or emotional problems

A paired sample *t* test evaluated change in parent-reported scores from baseline to week 12. The behavior changes were significant (see Fig. 1), t(2) = 23.00, *p* = .002, with a large effect size (*d* = 5.95).

**Fig. 2:**
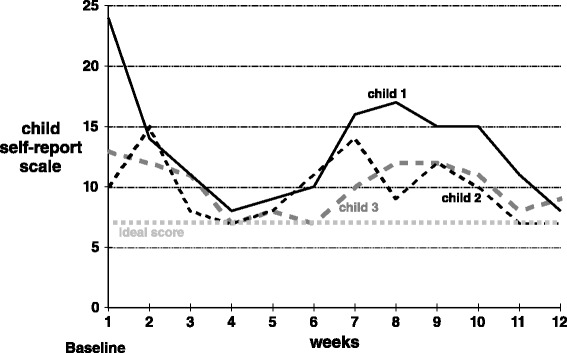
Child-reported scores. Each weekly data point is the sum of seven daily ratings, each of which could range from 1 to 4. Hence, a weekly score could range from 7 to 28, with 7 indicating that the child consistently reported feeling happy every day that week

There were no separate baseline data points for the children’s data, as the checklist began the same day the children began the nutrient formula, albeit at the initial low dose of only one capsule/day. Statistical testing of this scale was not appropriate, as it was a repeated measures nominal scale. Figure 2 should be interpreted as simply showing a trend toward the ideal score of 7 (which would reflect “happy” for all 7 days in a week).

On the parent questionnaire submitted weekly, there was no evidence of adverse events such as irritability or fatigue. The study coordinator’s biweekly in-person interview also did not uncover any reports of adverse events.

## Discussion

The primary purpose of this pilot work was to determine the feasibility of conducting a future, school-based evaluation of micronutrient treatment in children with emotional dysregulation and behavior problems. Feasibility was examined from several perspectives, and the result for each was quite positive: all three children remained in the trial throughout the 12 weeks, indicating that sample retention was not a problem; supplement adherence was excellent as all three families reported that they ensured their child took the daily doses; and the rating forms appeared to be easy for both parents and children to complete. As reported in many other studies, as well as in an article on safety and toxicity of this formula [9], no adverse reactions emerged. The measures used to monitor symptoms seemed to be sensitive to the changes reported verbally by parents in one-to-one interviews (reported below as “Parental perspectives”).

Although evaluating the actual treatment impact of this micronutrient formula on behavior was not the primary focus of this pilot feasibility work, the changes that emerged were very positive, as shown in Figs. 1 and 2. However, interpretation of those changes must be tempered by several limitations. The study employed no placebo and no teacher evaluations (as it was carried out during summer holidays). One could assume that a large positive expectancy effect would be possible, given this design.

## Conclusions

The primary conclusion of this pilot study is that further evaluation of micronutrient treatment in school-aged children with significant emotional and behavioral challenges is warranted and feasible. Such evaluations could be carried out both in the context of research as well as clinical use. Children with learning challenges warranting special settings such as the Learning Curve Academy have many obstacles to achievement; finding out whether just one set of obstacles (attention and behavior problems) can be ameliorated to enhance their educational experience is inherently worthwhile. It is this set of challenges which make it imperative that research be carried out in naturalistic, educational settings.

Health care personnel often struggle to help families cope with children’s emotional and attentional problems in school. Family physicians and pediatricians, in particular, are often confronted with the challenge of improving the lives of families who are burdened by school crises due to children’s emotional and behavioral dysregulation. The burden of these crises affects many people: the children themselves, their families, fellow classmates, and school personnel. The children described here, whose families volunteered to help determine the feasibility of further research at their school, experienced impressive changes that clearly warrant both research and clinical exploration. Currently, children’s weaknesses in attention and behavior control often result in medical interventions that may or may not be the ideal choice. There is significant potential for improved educational experiences of school-aged children who receive enhanced nutrition, which is a compelling reason to encourage further research.

### Parental perspectives

Language and cultural differences were barriers to obtaining written parental descriptions of their perspectives. Hence, information was obtained from personal interviews carried out by PH with the assistance of a translator who attended each meeting:

From the mother of child 1 (a 5-year-old boy):

- *After 4 weeks*: She no longer has to spank him; she can now communicate with him. As soon as she started giving him four capsules/day (week 4), she saw big changes. He goes to sleep quicker without a fight; he calms down quicker; his social and family time is improved because he is behaving now; more loving with his mother; used to annoy family members and is now caring; more independent and compassionate and sentimental.
- *After 6 weeks*: She said her son was never diagnosed with a mental disorder, but she had a referral to see a psychologist when she was introduced to [the nutrient formula]. Now she does not believe he needs to see the psychologist.
- *At the end of the trial*: The mother reported significantly improved behavior at home. She said he became calmer and slowed down. She said he was less hyper, more focused.

From the mother of child 2 (a 6-year-old boy).

- *After 4 weeks*: She reports that her son has become so relaxed, they often have to wake him up; he is sleeping much better and sleeping longer; much calmer; drinking more water; would rather drink water than Hawaiian Punch; less accident prone; sharing with his sister; seeks approval from his mother for good behavior; more sociable; family atmosphere improved because of better behavior.
- *At the end of the trial*: During the previous year this child lived in his own world. He chose not to communicate with his family and he had no friends. At around week 3 on the micronutrient formula, he started making friends and started being nicer to his sister. This child had also never shown emotion prior to the nutrient trial, but after 4 weeks on the micronutrients, his mother reported that she was crying one day, and her son came to her, put his arm around her, and said, “It’s OK Mommy, don’t cry.” This amount of compassion had not been exhibited by him prior to this time.

From the father of child 3 (a 14-year-old girl):

- *After 6 weeks*: The father reported she is sleeping so much better. She used to suffer from insomnia for 2–3 days straight. She was hyperactive 24 hours a day, 7 days a week. She talked really fast and changed subjects too quickly. She used to fight with everybody. Dad could not trust her; could never leave her alone. The father said his child herself does not remember back to before she was taking the nutrients; she does not remember how bad she was and shrieks in horror when she listens to her father describe how bad she was compared to how good she is now.
- *After 9 weeks*: They just moved again (during week 9) for the second time this summer, and life has been very hectic. By the third week, she was sleeping better and her behavior was improving. He told us that his daughter had been wanting an iPad for a long time, and he had continued to tell her “no” because her behavior was so bad. At the end of the trial he was pleased to report that he was able to buy her one. He also reported that her improved behavior had given him a lot of peace. The other people around this girl who knew her well (for example, the interpreter and the school principal) reported that it was a miracle how this child’s behavior had improved.

## Consent

Written informed consent was obtained from the patients’ legal guardians for publication of these three cases and any accompanying data. Copies of the written consents are available for review by the Editor-in-Chief of this journal.

## Footnotes

1 There are several variants of EMPowerplus Advanced with similar formulas, such as Truehope EMP, EMPowerplus, Q96, and Daily Essential Nutrients. The ingredients of the one used in these case reports can be found on the following website: www.Truehope.com

## References

[1] Schoenthaler SJ, Bier ID. The effect of vitamin-mineral supplementation on juvenile delinquency among American schoolchildren: a randomized, double-blind placebo-controlled trial. J Altern Complement Med. 2000;6:7–17. 10.1089/acm.2000.6.7 10706231

[2] Harding KL, Judah RD, Gant C. Outcome-based comparison of Ritalin versus food-supplement treated children with AD/HD. Altern Med Rev. 2003;8:319–30. 12946241

[3] Katz M, Levine AA, Kol-Degani H, Kav-Venaki L. A compound herbal preparation (CHP) in the treatment of children with ADHD: a randomized controlled trial. J Atten Disord. 2010;14:281–91. 10.1177/1087054709356388 20228219

[4] Kaplan BJ, Fisher JE, Crawford SG, Field CJ, Kolb B. Improved mood and behavior during treatment with a mineral-vitamin supplement: an open-label case series of children. J Child Adolesc Psychopharmacol. 2004;14:115–22. 10.1089/104454604773840553 15142398

[5] Rucklidge JJ, Gately D, Kaplan BJ. Database analysis of children and adolescents with bipolar disorder consuming a multinutrient formula. BMC Psychiatry. 2010;10:74. 10.1186/1471-244X-10-74 PMC2954995 20875144

[6] Frazier EA, Fristad MA, Arnold LE. Feasibility of a nutritional supplement as treatment for pediatric bipolar spectrum disorders. J Altern Complement Med. 2012;18:678–85. 10.1089/acm.2011.0270 22747095

[7] Rucklidge JJ, Frampton CM, Gorman B, Boggis A. Vitamin-mineral treatment of attention-deficit hyperactivity disorder in adults: double-blind randomised placebo-controlled trial. Br J Psychiatry. 2014;204:306–15. 10.1192/bjp.bp.113.132126 24482441

[8] Pavuluri MN, Henry DB, Devineni B, Carbray JA, Birmaher B. Child mania rating scale: development, reliability, and validity. J Am Acad Child Adolesc Psychiatry. 2006;45:550–60. 10.1097/01.chi.0000205700.40700.50 16601399

[9] Simpson JSA, Crawford SG, Goldstein ET, Field C, Burgess E, Kaplan BJ. Systematic review of safety and tolerability of a complex micronutrient formula used in mental health. BMC Psychiatry. 2011;11:62. 10.1186/1471-244X-11-62 PMC3094286 21501484

